# Treatment, Survival, and Demographics in Temporal Bone Malignancies: A Pooled Data Analysis

**DOI:** 10.7759/cureus.31973

**Published:** 2022-11-28

**Authors:** Mitchell R Gore

**Affiliations:** 1 Otolaryngology - Head and Neck Surgery, State University of New York Upstate Medical University, Syracuse, USA

**Keywords:** survival, oncology, head and neck, ear, cancer

## Abstract

Temporal bone malignancies are relatively uncommon tumors. Their location adjacent to vital structures such as the carotid artery, jugular vein, otic capsule, and temporal lobe can make their treatment potentially challenging. The purpose of this study was to compare outcomes in temporal bone malignancies obtained via pooled literature data.

The study sought to examine factors affecting survival in temporal bone malignancies based on the studies in the existing published literature. A systematic search was conducted on the PubMed (Medline), Embase, and Google Scholar databases to identify relevant studies from 1951 to 2022 that described the treatment of temporal bone malignancies. Articles that fulfilled the inclusion criteria were assessed and analyzed by the author.

The literature search identified 5875 case series and case reports, and 161 of them contained sufficient data to be included in the pooled data analysis, involving a total of 825 patients. Multivariate analysis of the pooled literature data showed that overall stage, presence of facial palsy, and surgical margin status significantly affected overall survival (OS), while overall stage and presence of facial palsy significantly affected disease-free survival (DFS).

To summarize, this study examined pooled survival data on demographics, treatment, and survival of patients with temporal bone malignancies utilizing an extensive literature-based pooled data meta-analysis. Overall stage, facial nerve status, and surgical margin status appeared to most strongly affect survival in patients with temporal bone malignancies.

## Introduction and background

The temporal bone is a relatively rare site of malignancy. Ear canal or middle ear cancers affect approximately one patient per million each year, and temporal bone cancers account for approximately only 0.2% of all head and neck cancers [1]. These patients tend to be older, especially patients with squamous cell carcinoma of the temporal bone. The common symptoms can include otalgia, otorrhea, hearing loss, and more ominously, facial palsy. Squamous cell carcinoma is typically the most common histopathological type found in temporal bone cancer. Surgery is the mainstay of treatment, with adjuvant radiation provided in addition to surgery for advanced disease. Patient survival is typically lower in patients with tumors of a higher stage and higher grade, those with positive surgical margins, and locoregional or distant spread [1]. Given the relative rarity of temporal bone malignancies, a pooled data analysis of cases reported in the literature may be a useful tool for studying patient and tumor characteristics affecting survival in these patients. Hence, this study sought to evaluate patient demographics and survival outcomes for patients with temporal bone malignancies documented in the literature from 1951 to 2022 and to analyze the demographics, treatment, and survival data found therein.

## Review

Review of the literature

Using the Preferred Reporting Items for Systematic Reviews and Meta-Analyses (PRISMA) criteria, a systematic search of the PubMed (Medline), Embase, and Google Scholar databases was conducted [2]. The following search terms were employed: “temporal bone + cancer + malignant + carcinoma”. The Newcastle-Ottawa Quality Assessment Scale for Case-Control Studies was utilized to assess the included studies [3]. Studies were included if they were published in any language in scientific journals published in the PubMed (Medline), Embase, and Google Scholar databases, and reported data on human subjects with newly diagnosed and previously untreated primary solid temporal bone malignancies (hematologic malignancies, metastases to the temporal bone or local spread to the temporal bone from primary parotid malignancies, and recurrent tumors previously treated were excluded), with sufficient individual patient data on overall survival (OS) and disease-free survival (DFS).

**Figure 1:**
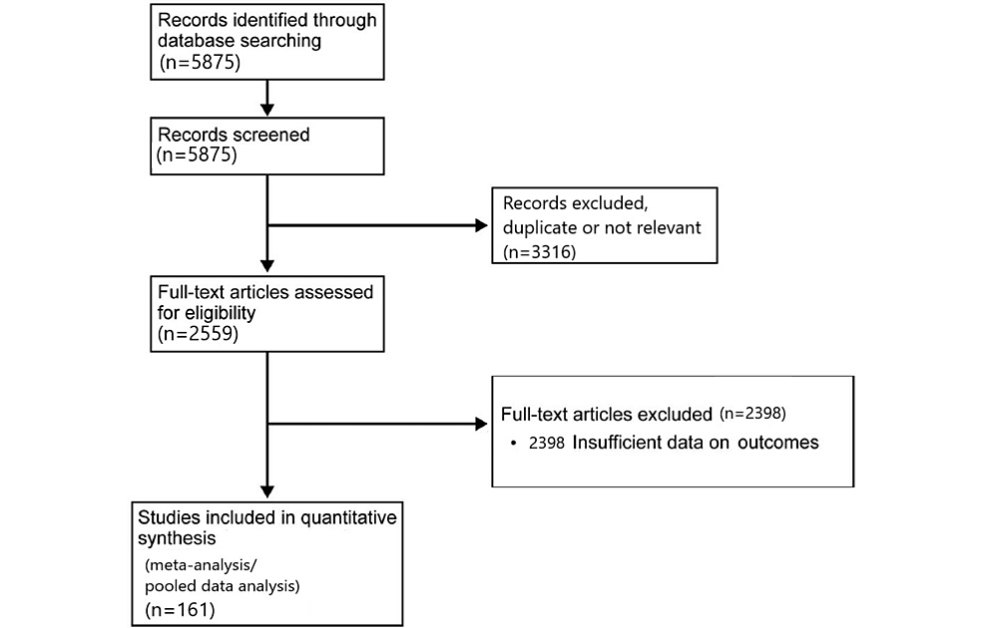
PRISMA study selection flow diagram for the systematic literature search PRISMA: Preferred Reporting Items for Systematic Reviews and Meta-Analyses

The literature search was conducted from February 1 to 28, 2022. The titles and abstracts of the articles were reviewed and articles with duplicate or irrelevant data were excluded. The remaining articles were reviewed in full by the author and articles with sufficient and relevant individual patent data on temporal bone malignancies were included in the pooled data analysis. The individual patient data on OS and DFS, overall and tumor (T), nodal (N), and metastasis (M) stages, solid tumor type, age at diagnosis, treatment type, surgery type if applicable, presence of hearing loss and presence of facial nerve palsy at diagnosis, and surgical margin status if applicable were compiled into a Microsoft Excel spreadsheet (Microsoft Corp., Redmond, Washington, USA) for the 161 studies included in the pooled data analysis [4-165]. The search for studies that spanned the period from 1951 to 2022 yielded a total of 5875 articles. After excluding articles not related to primary human temporal bone malignancies (animal studies, basic science/in vitro studies, etc.), 2559 articles were assessed for the availability of individual patient data. Applying these exclusion criteria yielded 161 articles with individual patient data on primary temporal bone malignancies involving 835 patients, 825 of whom had full analyzable survival data. The data were compiled and checked by the author for accuracy. Figure 1 illustrates the PRISMA selection process for the literature pooled data analysis.

The staging was performed using the Modified Pittsburgh Staging System for temporal bone malignancies [165,166]. The primary survival outcomes were OS and DFS at 60 months. Survival outcomes were analyzed using the Kaplan-Meier/log-rank method. Statistical analysis was performed using XLSTAT Biomed (Addinsoft, New York, NY/Paris, France). Linear regression was used to perform multivariate analysis. A p-value of less than 0.05 was considered statistically significant. Fisher’s exact test was used to determine odds ratios.

Results

Pooled Data Meta-Analysis: Demographics

The pooled data meta-analysis identified 161 studies with individual patient data involving 835 patients, with 825 having analyzable OS and DFS data. Patient sex was unknown in 333 of 825 (40.4%) patients, while 237 (28.7%) were female and 255 (30.9%) were male. The average patient age was 54 ±23 years. Of the 825 patients with analyzable survival data, 621 had squamous cell carcinoma (75.2%), 126 had sarcoma (15.3%), 27 had adenocarcinoma (3.3%), 19 had basal cell carcinoma (2.3%), nine had mucoepidermoid carcinoma (1.1%), 10 had adenoid cystic carcinoma (1.2%), and 13 had melanoma (1.6%).

Of the 825 patients with analyzable survival data, there were 113 stage I (13.7%), 88 stage II (10.7%), 210 stage III (25.5%), 342 stage IV (41.5%), and 72 unknown stage patients (8.7%). There were 167 T1 (20.2%), 150 T2 (18.2%), 190 T3 (23.0%), 277 T4 (33.6%), and 41 unknown T stage (5.0%) patients. The cohort included 608 N0 (73.7%), 55 N1 (6.7%), 40 N2 (4.8%), 5 N3 (0.6%), and 117 unknown N stage (14.2%) patients. There were 656 M0 (79.5%), 55 M1 (6.7%), and 114 unknown M stage (13.8%) patients.

Pooled Data Meta-Analysis: Patient Presentation

In 661 of 825 patients (80.1%), hearing loss status was unknown, while in the 164 (19.9%) patients whose hearing loss status was known, 109 had hearing loss (66.5%) and 55 did not have hearing loss (33.5%). Facial nerve status was unknown in 564 of 825 patients (68.4%). Of the remaining 261 patients (31.6%) in whom the presence or absence of facial palsy was specified, 107 (41.0%) had some degree of facial palsy while 154 (59.0%) had normal facial nerve function.

Pooled Data Meta-Analysis: Treatment and Pathology

Surgical margin status was known in 313 of 825 patients (37.9%); 131 of 313 had positive margins (41.9%) and 182 had negative margins (58.1%). The remaining patients were either treated nonsurgically (203, 24.6%), or their margin status was unknown (309, 37.5%). Tumor grade was unknown in 728 of 825 patients (88.2%), low grade/well-differentiated in 47 (5.7%), intermediate grade/moderately differentiated in 28 (3.4%), and high grade/poorly differentiated in 22 (2.7%). Of the patients for whom the treatment type was known, 59 (7.2%) were treated with surgery + radiation + chemotherapy, 330 (40.0%) were treated with surgery + radiation, 90 (10.9%) were treated with radiation + chemotherapy, 21 (2.5%) with surgery + chemotherapy, five (0.6%) with chemotherapy alone, 155 (18.8%) with surgery alone, and 98 (11.9%) with radiation alone. The treatment type was none in 10 patients (1.2%) and unknown in the remaining 57 patients (6.9%).

**Figure 2:**
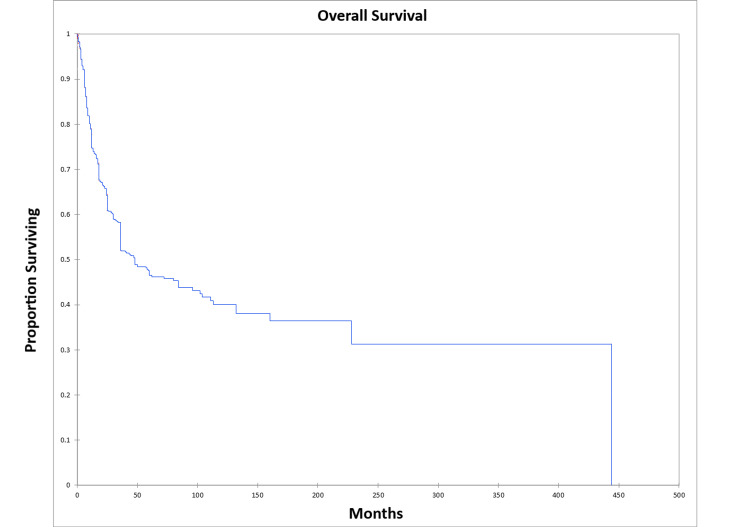
Kaplan-Meier overall survival for the entire literature-based temporal bone malignancy cohort

**Figure 3:**
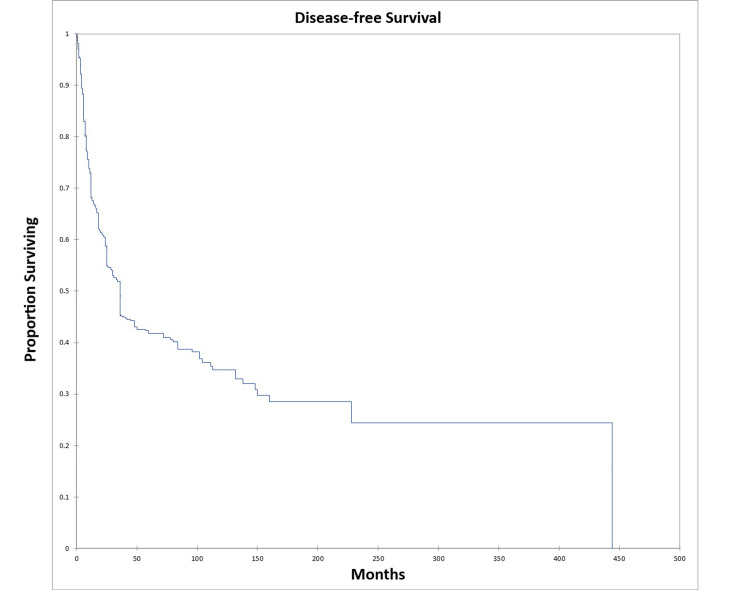
Kaplan-Meier disease-free survival for the entire literature-based temporal bone malignancy cohort

OS at five and 10 years was 46.0% and 39.0% respectively for the entire cohort. DFS at five and 10 years was 41.0% and 34.0% respectively for the entire cohort. Figure 2 shows the Kaplan-Meier OS for the entire cohort, while Figure 3 shows the Kaplan-Meier DFS for the entire cohort.

**Figure 4:**
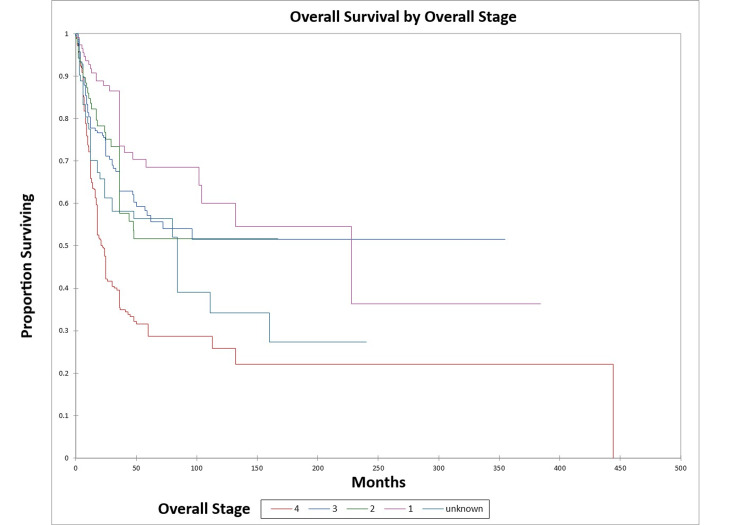
Kaplan-Meier overall survival by overall stage for literature-based temporal bone malignancy cohort

**Figure 5:**
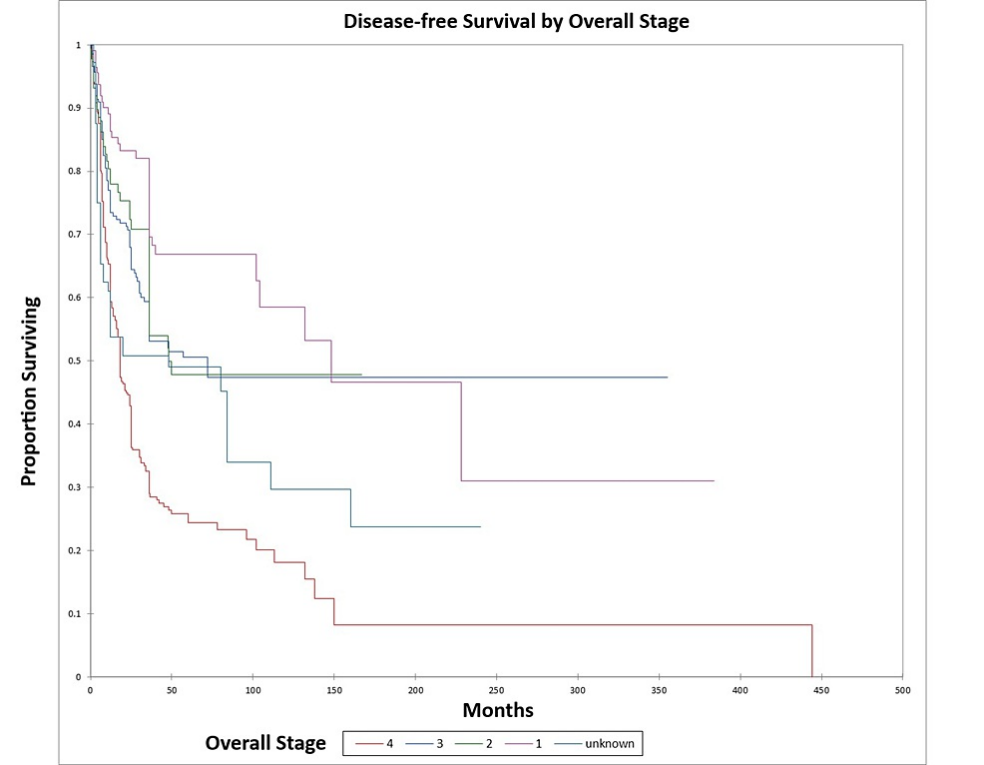
Kaplan-Meier disease-free survival by overall stage for literature-based temporal bone malignancy cohort

Figure 4 and Figure 5 illustrate the Kaplan-Meier OS (p<0.0001) and DFS (p<0.0001) respectively by overall stage.

**Figure 6:**
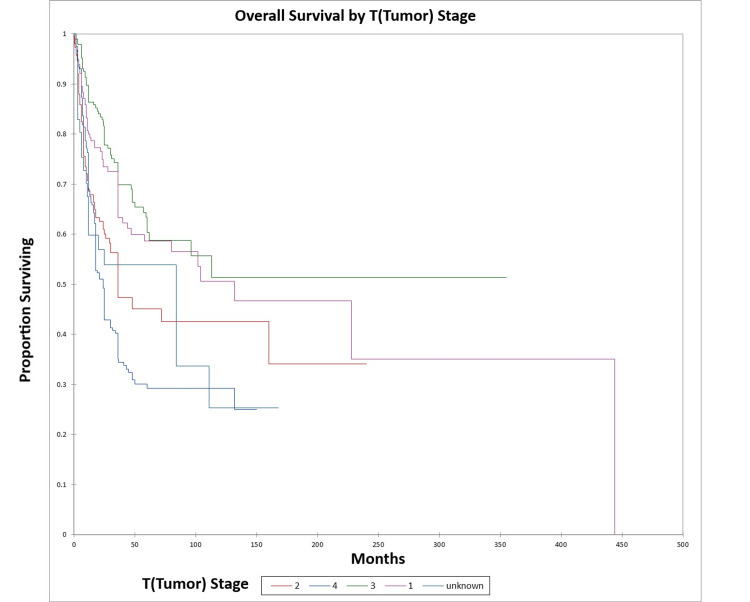
Kaplan-Meier overall survival by T (tumor) stage for literature-based temporal bone malignancy cohort

**Figure 7:**
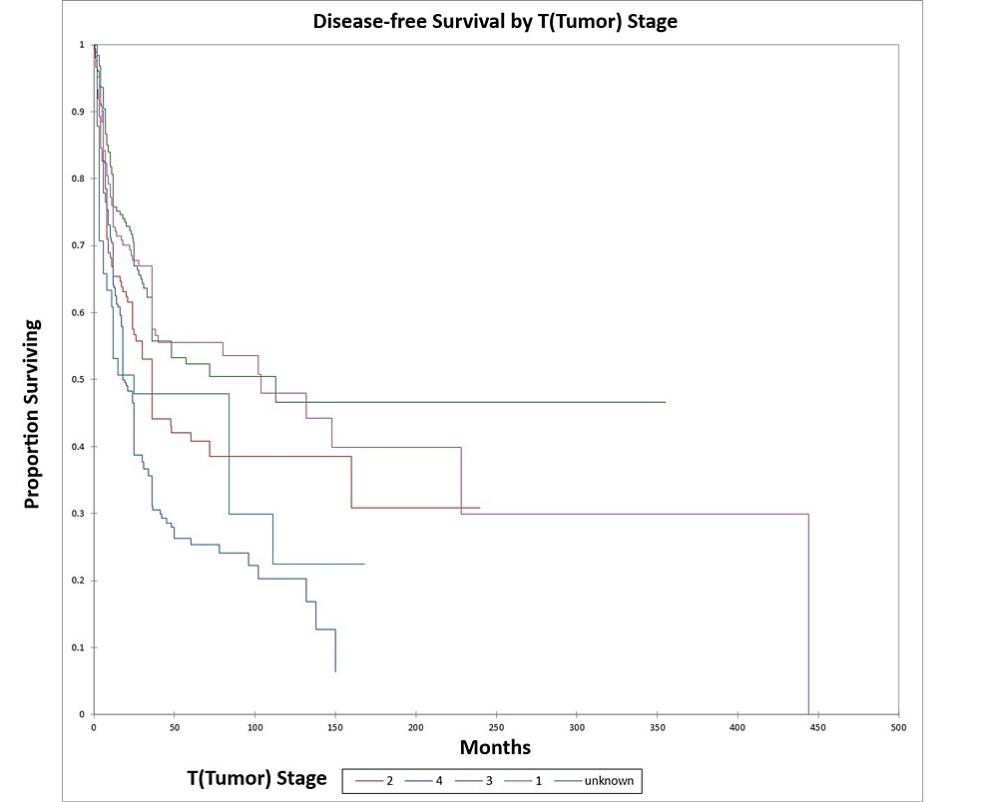
Kaplan-Meier disease-free survival by T (tumor) stage for literature-based temporal bone malignancy cohort

Figure 6 and Figure 7 illustrate the Kaplan-Meier OS (p<0.0001) and DFS (p<0.0001) respectively by T (tumor) stage.

**Figure 8:**
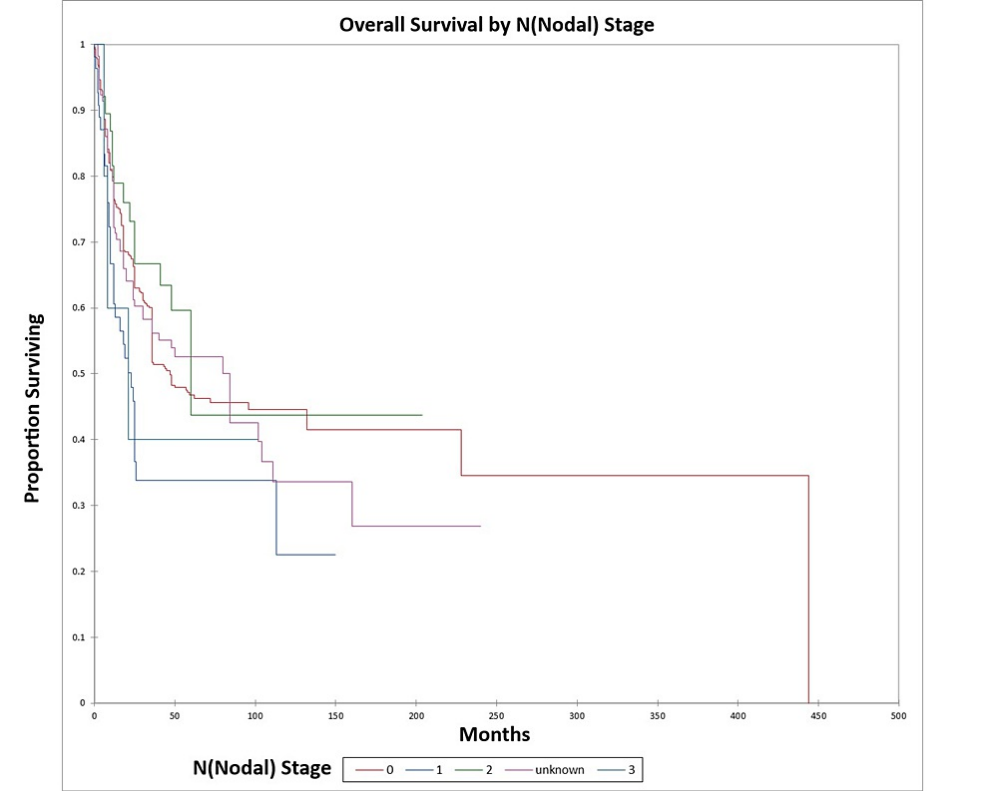
Kaplan-Meier overall survival by N (nodal) stage for literature-based temporal bone malignancy cohort

**Figure 9:**
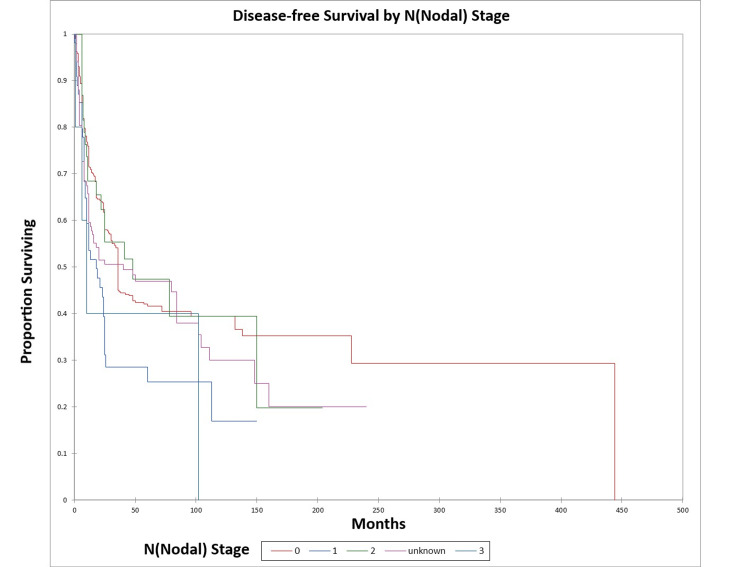
Kaplan-Meier disease-free survival by N (nodal) stage for literature-based temporal bone malignancy cohort

Figure 8 and Figure 9 illustrate the Kaplan-Meier OS (p=0.04) and DFS (p=0.01) respectively by N (nodal) stage.

**Figure 10:**
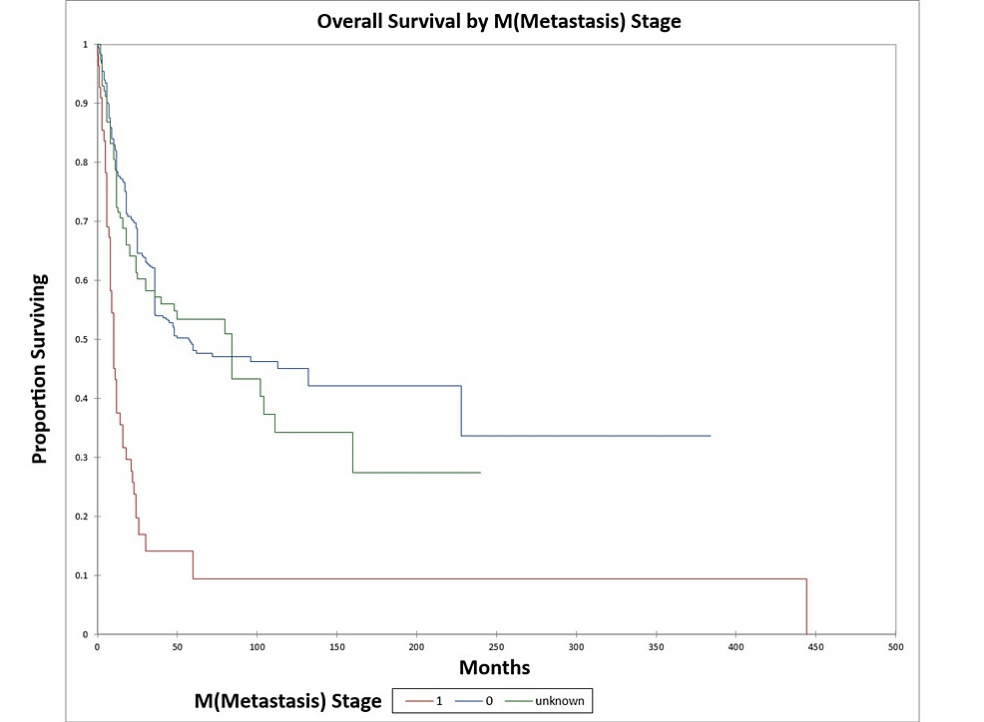
Kaplan-Meier overall survival by M (metastasis) stage for literature-based temporal bone malignancy cohort

**Figure 11:**
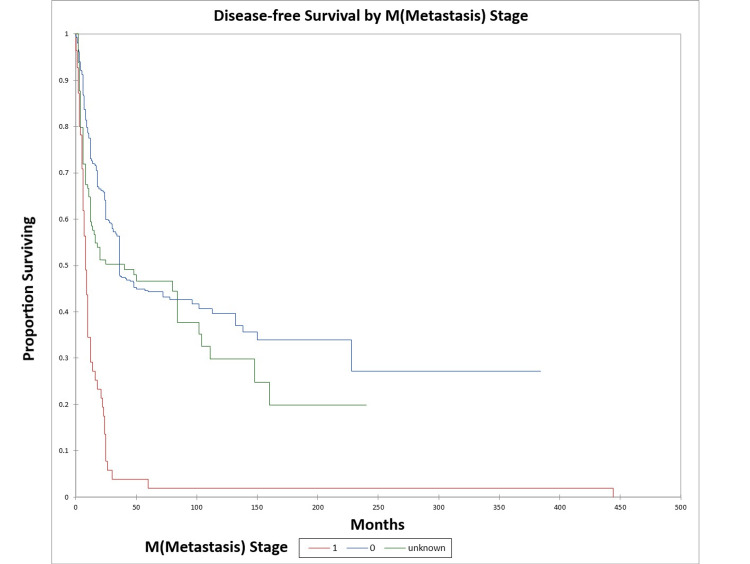
Kaplan-Meier disease-free survival by M (metastasis) stage for literature-based temporal bone malignancy cohort

Figure 10 and Figure 11 illustrate the Kaplan-Meier OS (p<0.0001) and DFS (p<0.0001) respectively by M (metastasis) stage. The odds ratio for death at five years for M1 patients vs. M0 patients was 9.6 [95% confidence interval (CI): 3.8-24.3) (OS)], and 44.1 (95% CI: 6.1-321.0) (DFS).

**Figure 12:**
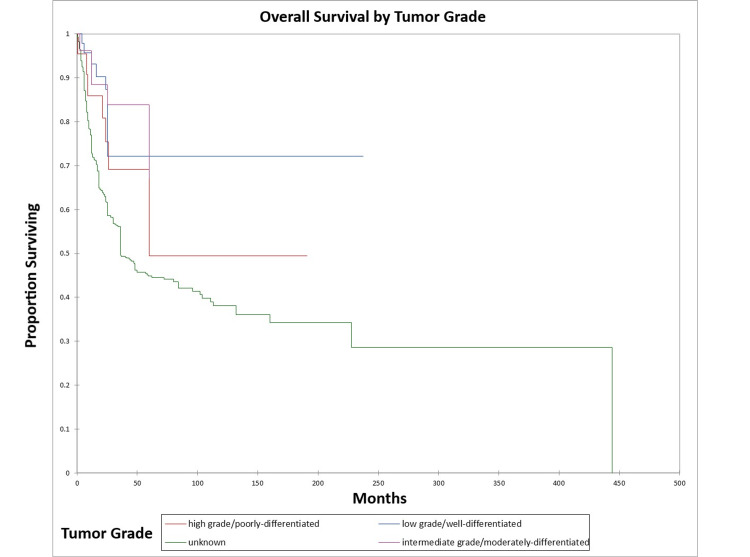
Kaplan-Meier overall survival by tumor grade for literature-based temporal bone malignancy cohort

**Figure 13:**
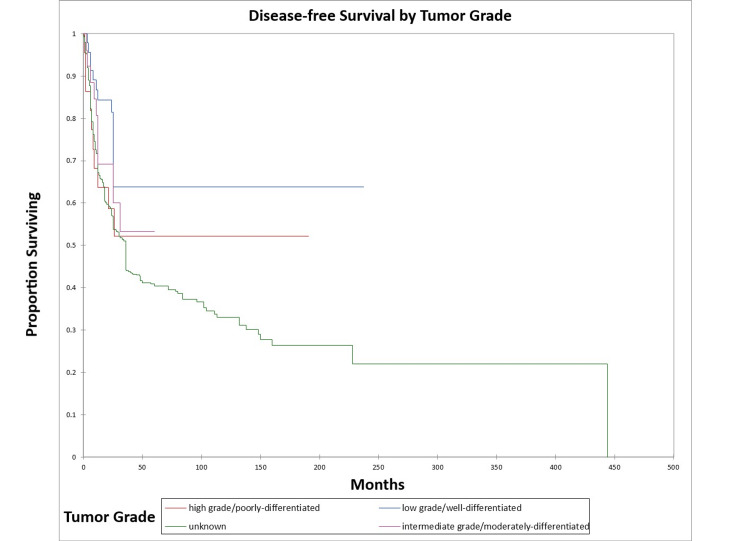
Kaplan-Meier disease-free survival by tumor grade for literature-based temporal bone malignancy cohort

Figure 12 and Figure 13 illustrate the Kaplan-Meier OS (p=0.002) and DFS (p=0.06) respectively by tumor grade.

**Figure 14:**
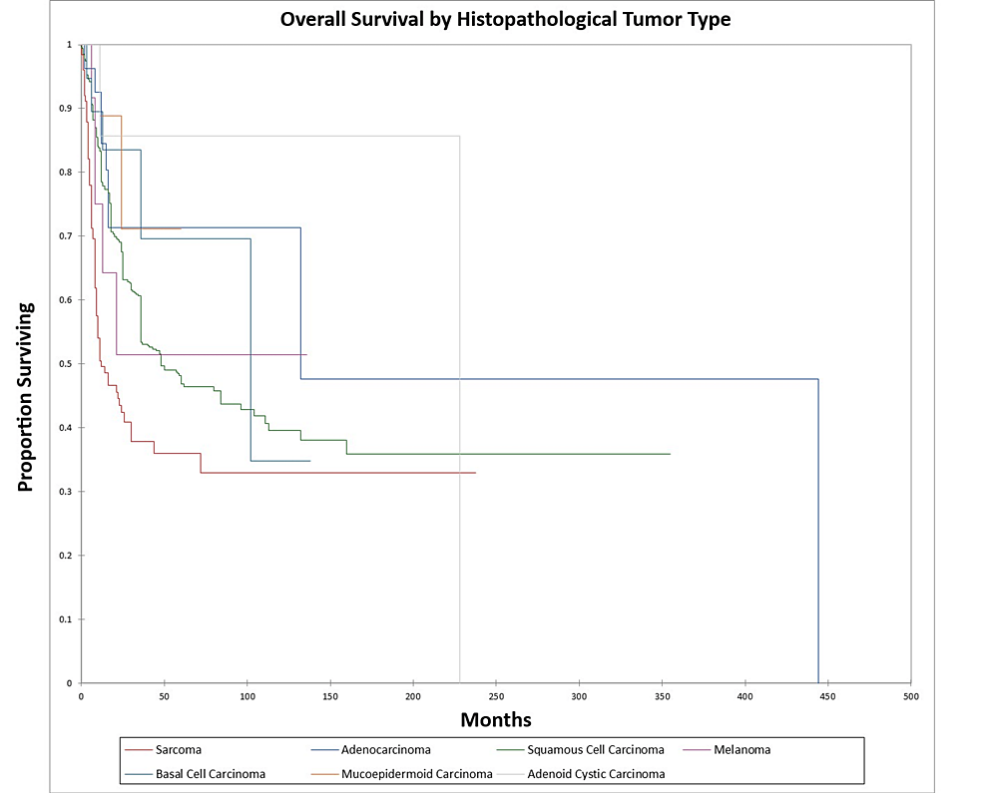
Kaplan-Meier overall survival by histopathological type for literature-based temporal bone malignancy cohort

**Figure 15:**
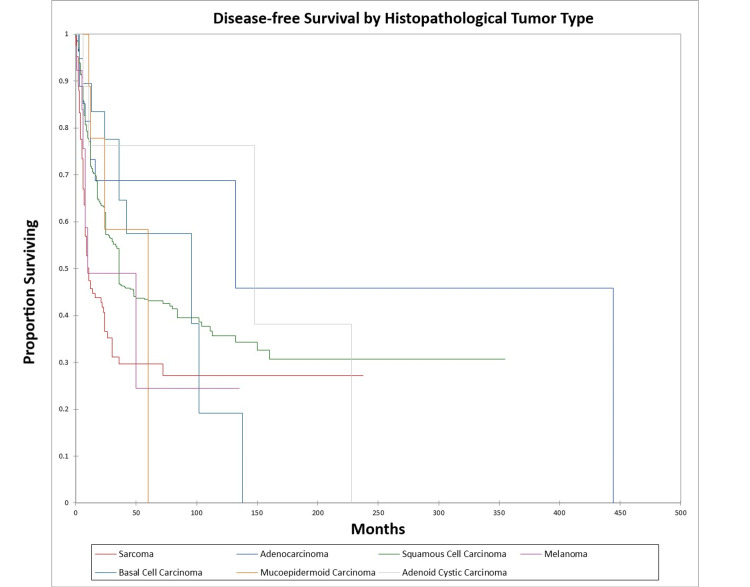
Kaplan-Meier disease-free survival by histopathological type for literature-based temporal bone malignancy cohort

Figure 14 and Figure 15 illustrate the Kaplan-Meier OS (p<0.0001) and DFS (p<0.0001) respectively by histopathological tumor type. OS and DFS were highest for adenocarcinoma, adenoid cystic carcinoma, and basal cell carcinoma, and lowest for squamous cell carcinoma, sarcoma, mucoepidermoid carcinoma, and melanoma.

**Figure 16:**
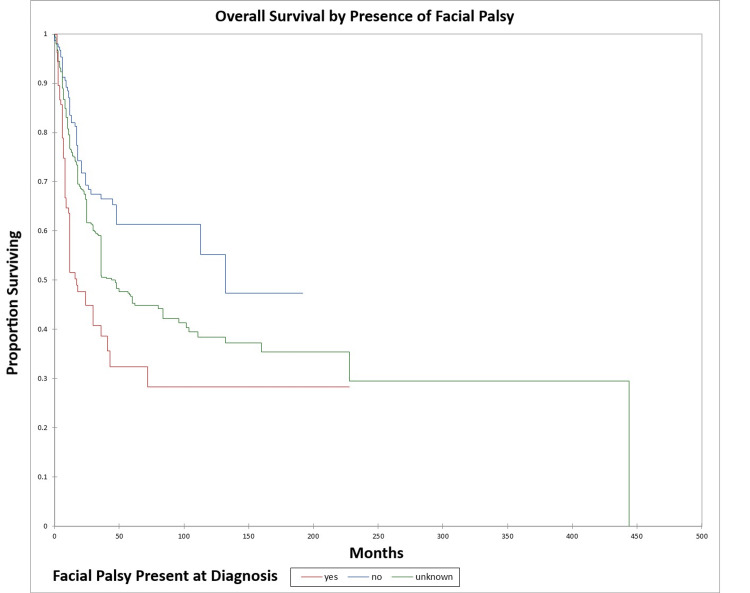
Kaplan-Meier overall survival by the presence or absence of facial palsy for literature-based temporal bone malignancy cohort

**Figure 17:**
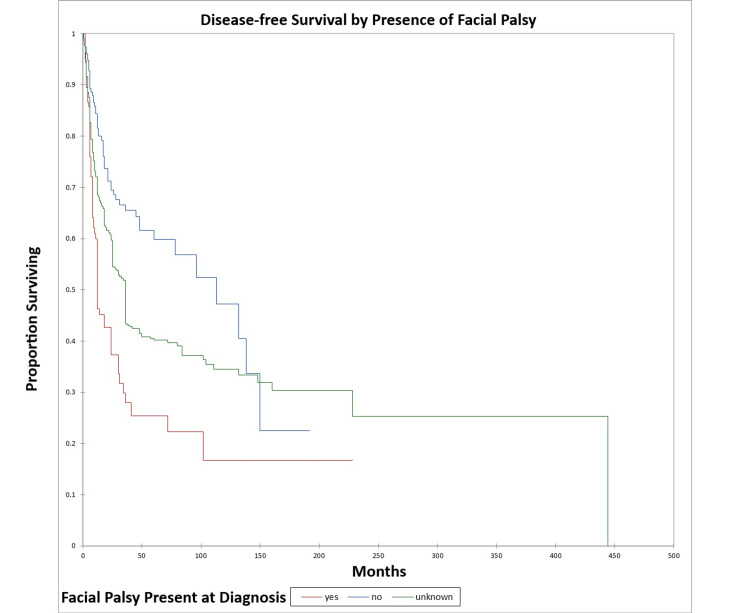
Kaplan-Meier disease-free survival by the presence or absence of facial palsy for literature-based temporal bone malignancy cohort

Figure 16 and Figure 17 illustrate the Kaplan-Meier OS (p<0.0001) and DFS (p<0.0001) respectively by the presence or absence of facial nerve palsy. The odds ratio for death at five years for patients with facial palsy vs. patients without facial palsy was 3.5 (95% CI: 2.1-5.8) (OS) and 4.3 (95% CI: 2.5-7.4) (DFS).

**Figure 18:**
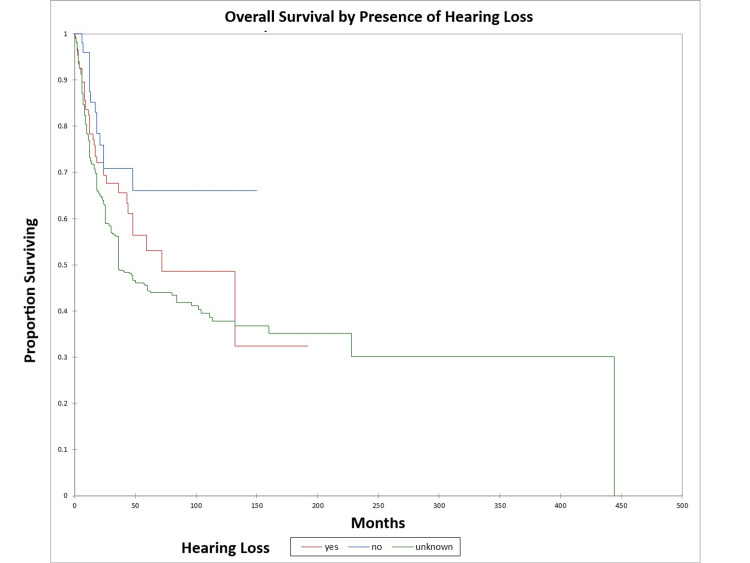
Kaplan-Meier overall survival by the presence or absence of hearing loss for literature-based temporal bone malignancy cohort

**Figure 19:**
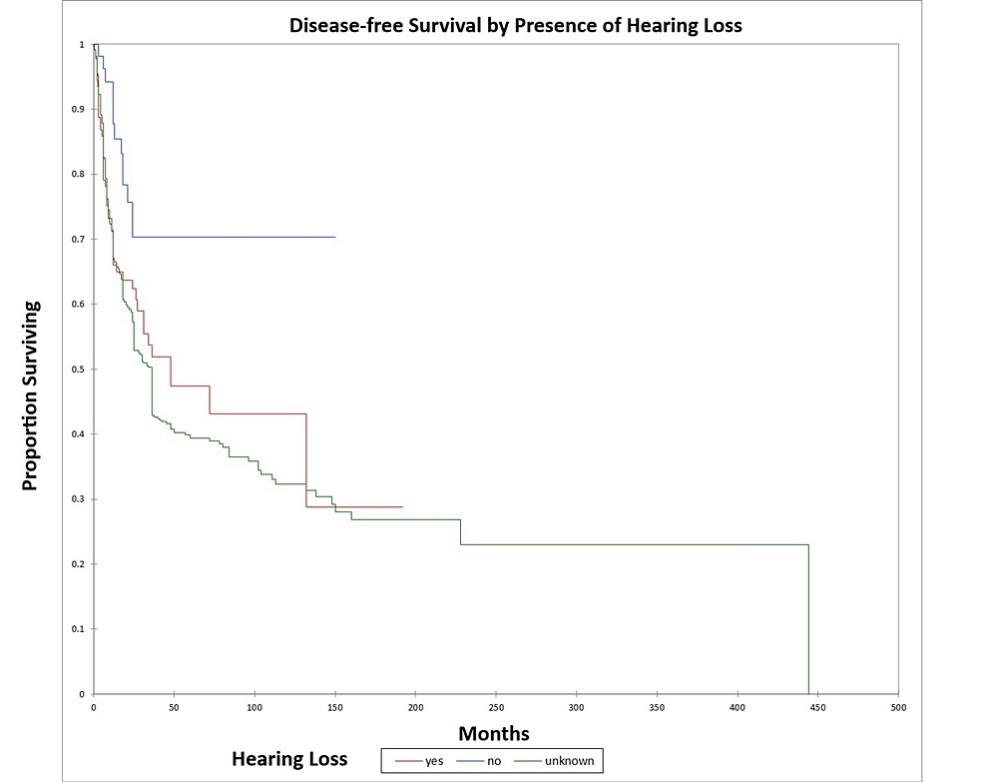
Kaplan-Meier disease-free survival by the presence or absence of hearing loss for literature-based temporal bone malignancy cohort

Figure 18 and Figure 19 illustrate the Kaplan-Meier OS (p=0.02) and DFS (p=0.003) respectively by the presence or absence of hearing loss. The odds ratio for death at five years for patients with hearing loss vs. patients with normal hearing was 1.73 (95% CI: 0.9-3.4) (OS) and 2.8 (95% CI: 1.4-5.5) (DFS).

**Figure 20:**
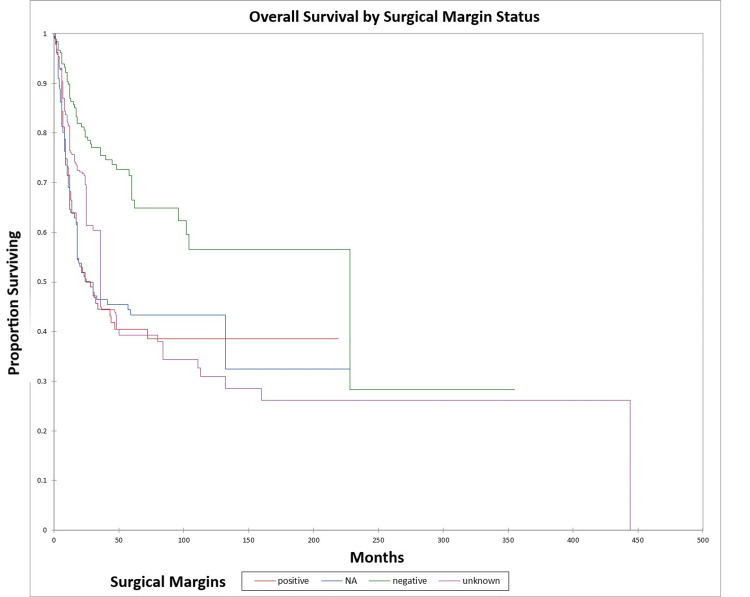
Kaplan-Meier overall survival by margin status for literature-based temporal bone malignancy cohort NA: not applicable

**Figure 21:**
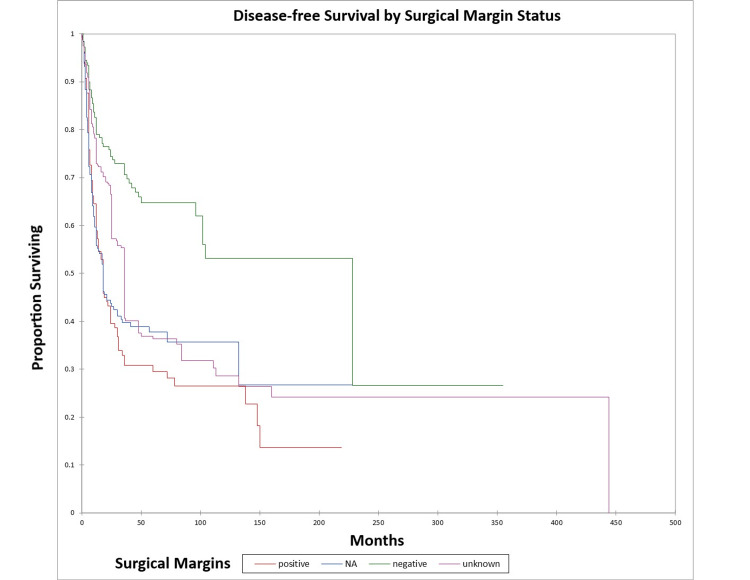
Kaplan-Meier disease-free survival by margin status for literature-based temporal bone malignancy cohort NA: not applicable

Figure 20 and Figure 21 illustrate the Kaplan-Meier OS (p<0.0001) and DFS (p<0.0001) respectively by margin status. The odds ratio for death at five years for patients with positive margins vs. patients with negative margins was 3.7 (95% CI: 2.3-5.9) (OS) and 4.5 (95% CI: 2.7-7.3) (DFS).

**Figure 22:**
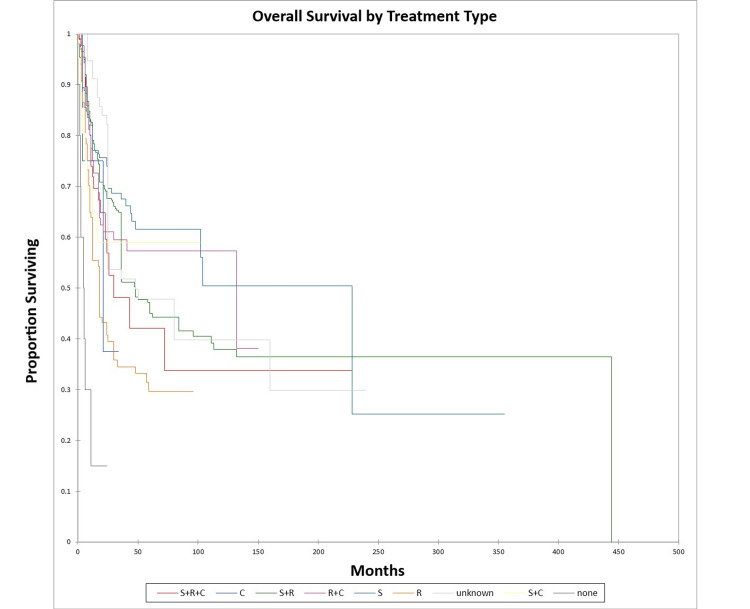
Kaplan-Meier overall survival by treatment type for literature-based temporal bone malignancy cohort S: surgery; R: radiation; C: chemotherapy

**Figure 23:**
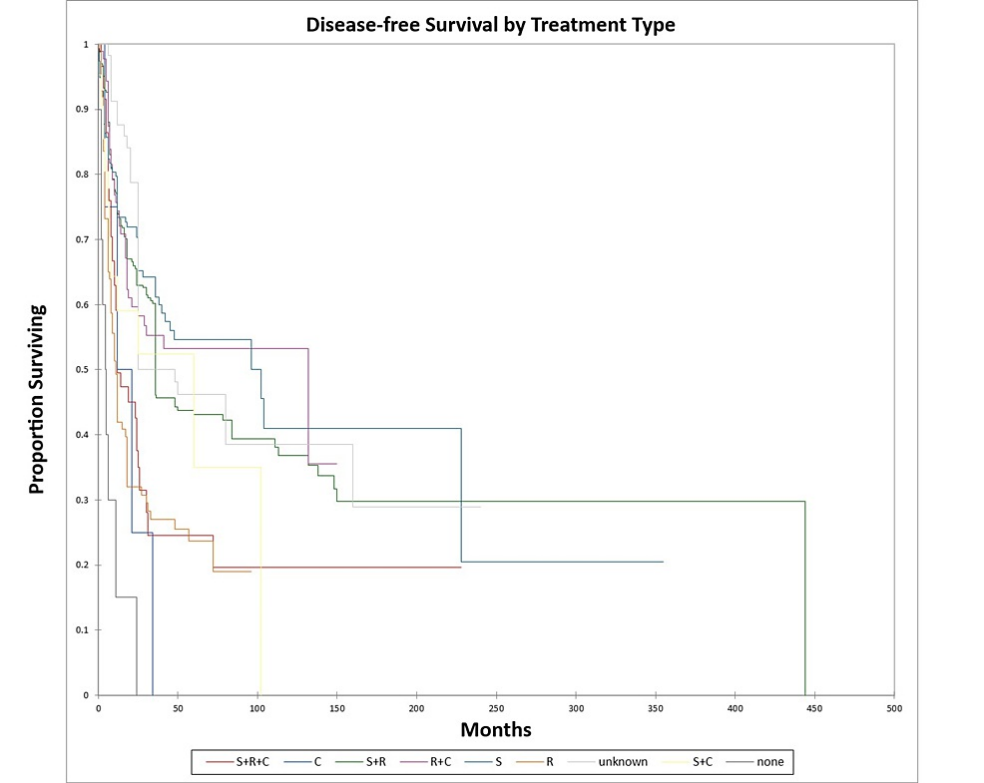
Kaplan-Meier disease-free survival by treatment type for literature-based temporal bone malignancy cohort S: surgery; R: radiation; C: chemotherapy

Figure 22 and Figure 23 illustrate the Kaplan-Meier OS (p<0.0001) and DFS (p<0.0001) respectively by treatment type.

**Figure 24:**
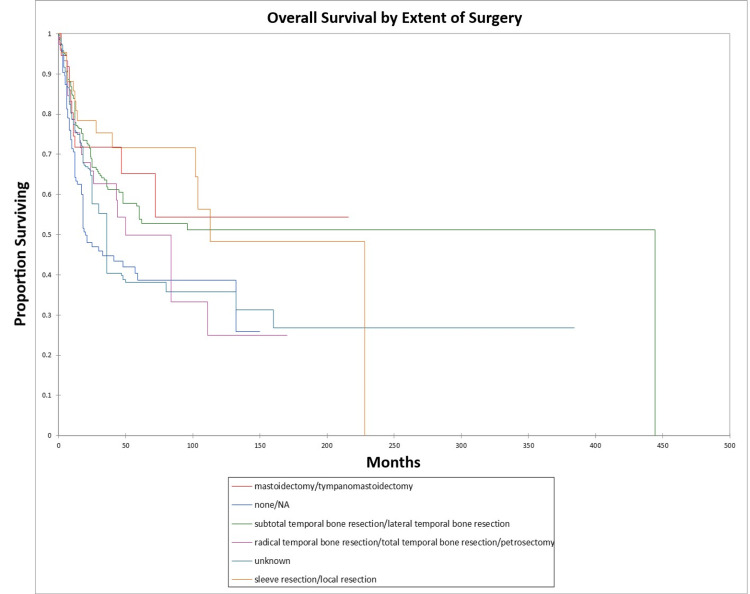
Kaplan-Meier overall survival by type of surgery for literature-based temporal bone malignancy cohort

**Figure 25:**
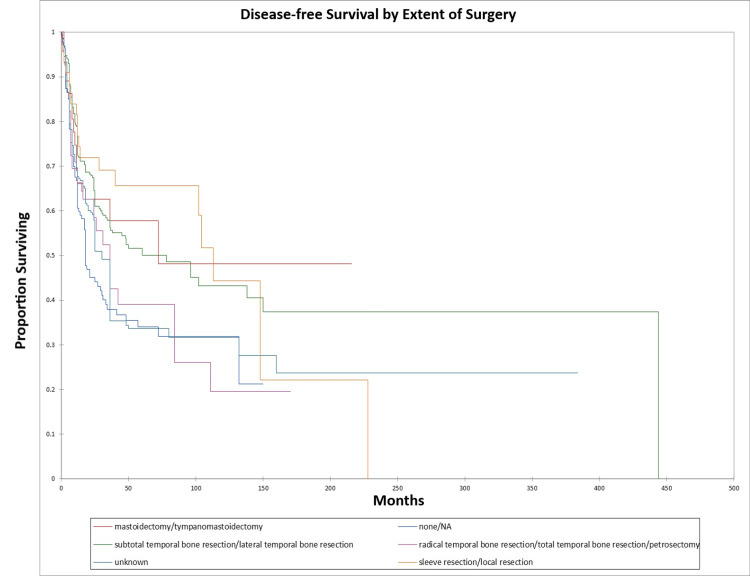
Kaplan-Meier disease-free survival by type of surgery for literature-based temporal bone malignancy cohort

Figure 24 and Figure 25 illustrate the Kaplan-Meier OS (p=0.0002) and DFS (p=0.0003) respectively by type of surgery performed.

Multivariate analysis demonstrated that only overall stage (p<0.0001), the presence or absence of facial palsy (p<0.0001), and surgical margin status (p=0.04) significantly affected OS, and only overall stage (p<0.0001) and the presence or absence of facial palsy (p=0.002) significantly affected DFS.

This study examined data on temporal bone malignancies based on a pooled data analysis of the literature on temporal bone cancers from 1951 to 2022. OS at five years was 46.0% for the entire literature-based cohort. The study included roughly equal numbers of male and female patients [sex unknown for 333/825 (40.4%) patients, while 237/825 were female (28.7%) and 255/825 were male (30.9%)]. The average patient age was 54 ±23 years. Squamous cell carcinoma was by far the most common histopathological type [621 of 825 patients (75.2%)], followed by sarcoma (126/825, 15.3%). Patients tended to present at a relatively advanced stage with 210/825 (25.5%) being stage III and 342/825 being stage IV (41.5%) at presentation, while only 113/825 (13.7%) were stage I and only 88/825 (10.7%) were stage II. The stage at presentation was unknown in 72/825 (8.7%). Nodal metastasis was relatively uncommon, with the cohort including 608/825 N0 (73.7%) patients and 100/825 (12.1%) N+ patients, with N stage unknown in 117/825 (14.2%) patients. Distant metastasis was also relatively uncommon with 656/825 (79.5%) M0 patients, 55/825 (6.7%) M1 patients, with M stage unknown in 114/825 (13.8%) patients. Patients treated with surgery alone (61.0% five-year OS/55.0% five-year DFS) had similar survival to patients treated with radiation + chemotherapy (58.0% five-year OS/53% five-year DFS) and patients treated with surgery + chemotherapy (59% five-year OS/52% DFS). Survival was somewhat lower for patients treated with radiation alone (32% five-year OS/25% five-year DFS) and patients treated with surgery, radiation, and chemotherapy (42% five-year OS/25% five-year DFS). This difference was significant on univariate analysis but not multivariate analysis. Treatment differences vary based on protocols at different centers, surgeon preference, and patient status/preference.

In the cohort, only the overall stage, presence or absence of facial palsy, and margin status significantly affected OS on multivariate analysis, while the overall stage and presence or absence of facial palsy significantly affected DFS. Survival was higher for adenocarcinoma and adenoid cystic carcinoma patients and lower for squamous cell carcinoma, melanoma, and sarcoma patients, a difference that was significant on univariate analysis but not multivariate analysis. The finding that facial nerve palsy significantly affects survival even on multivariate analysis confirms the results of the Pittsburgh group that patients with facial nerve palsy have survival patterns that most closely align with the T4 group [165-167]. The overall stage was a significant predictor of OS and DFS on multivariate analysis. Lechner et al. similarly noted that stage was a significant predictor of survival in their literature-based analysis of patients with squamous cell carcinoma of the temporal bone [168]. The finding that negative margins significantly improve OS on multivariate analysis is of note and unsurprising. The study by Wierzbicka et al. in 2017 also noted that positive margins significantly affected survival in temporal bone cancer patients [169]. Seligman et al. found that patients with temporal bone carcinoma had improved survival when patients had basal cell carcinoma, lateral temporal bone resection as opposed to subtotal temporal bone resection, were immunocompetent, and had no evidence of perineural or lymphovascular invasion [155]. Interestingly, in the present study, basal cell carcinoma patients had better OS and DFS than patients with squamous cell carcinoma at five years, but not at 10 years (69.0% and 34% five-year and 10-year OS and 58.0% and 19.0% five-year and 10-year DFS for basal cell carcinoma vs. 45.0% and 39.0% five-year and 10-year OS and 43.0% and 35.0% five-year and 10-year DFS for squamous cell carcinoma). This difference was significant on univariate but not multivariate analysis in the present study. Additionally, in the present study patients with adenocarcinoma and adenoid cystic carcinoma had better OS and DFS than basal cell carcinoma or squamous cell carcinoma patients on univariate analysis.

In the present study, subtotal temporal bone resection was grouped with lateral temporal bone resection in the statistical analysis. In general, at time points less than 120 months, patients who had sleeve/local resection or mastoidectomy had better survival than patients who underwent no surgery, patients who underwent subtotal or lateral temporal bone resection, or patients who underwent radical or total temporal bone resection or petrosectomy. The extent of resection may act as a surrogate for stage and/or resectability and may greatly be affected by surgeon preference, patient health/comorbidities, or differences in surgical technique at various centers [124]. In the present study, survival differences by the extent of surgery/surgery type were significant on univariate but not multivariate analysis. That extent of resection may act as a surrogate for the stage is borne out by the finding that in the present study, local excision/sleeve resection was essentially only used for T1/stage I patients. Shiga et al. retrospectively examined 23 patients treated for cancer of the temporal bone using concomitant chemoradiotherapy from 2001 to 2014 using docetaxel, cisplatin, and 5-fluorouracil in addition to radiotherapy [7]. They noted disease-specific five-year survival rates of 84.9% for the entire cohort, 100% for stage I, II, and III patients, and 75.5% for stage IV patients. Five-year survival in the present study was lower than the Shiga study for the entire literature cohorts, which may reflect the difference in sample size, or advances in treatment and survival in the years since the earlier patients treated in the literature cohort vs. the much more recent patients included in the Shiga study [7].

Nam et al. examined 26 patients with squamous cell carcinoma of the external auditory canal [170]. Similar to the present study, they noted that the T stage and overall stage significantly affected survival. The present study emphasizes the importance of accurate staging and, in patients treated surgically, negative surgical margins whenever possible. Acharya et al. examined The Surveillance, Epidemiology, and End Results (SEER) database for cases of carcinomas of the middle ear between 1975 and 2016, while Gurgel et al. examined the SEER database for patients diagnosed with middle ear carcinoma between 1973 and 2004 [171,172]. The Gurgel et al. study noted that the five-year OS rate for 215 patients with middle ear cancer was 36.4%, while the five-year overall survival rate for T3 patients (which would include patients with middle ear cancer) in the present study was 63% [172]. Interestingly, similar to the present study, the Gurgel study observed that patients with adenocarcinoma had improved OS vs. other histopathological types [172]. Squamous cell carcinoma was the most common pathological subtype identified in the present study, consistent with squamous cell carcinoma being the most common pathological subtype seen in head and neck cancer patients [173].

This study has several limitations. The retrospective nature of the literature-based cohort makes selection and recall bias a possibility. Additionally, the fact that the period studied spanned more than 50 years (1951-2022) means that the study had to incorporate various differences in treatment preferences over time as treatment protocols and technology evolved. Additionally, the cohort comprised patients from several different countries, which may also introduce differences in treatment preferences, patient demographics, etc. However, despite these shortcomings, the very granular data drawn from the cohort (facial nerve and hearing loss status, surgical margin status, histopathological type, etc.) is highly invaluable and allows for a detailed retrospective analysis of the patient factors and their relationship with OS and DFS in the patients in the cohort.

## Conclusions

This study retrospectively examined data on demographics, treatment, and survival of patients with temporal bone malignancies as per a literature-based pooled data analysis. Of note, overall stage and facial nerve status were significant predictors of OS and DFS on multivariate analysis, and surgical margin status significantly affected OS on multivariate analysis. Patient stage (overall and T, N, and M individual stages), surgical margin status, facial nerve palsy, hearing loss, treatment type, and histopathological type all appeared to significantly impact OS and DFS on univariate analysis.
